# Extended Analysis of Raman Spectra Using Artificial Intelligence Techniques for Colorectal Abnormality Classification

**DOI:** 10.3390/jimaging9120261

**Published:** 2023-11-24

**Authors:** Dimitris Kalatzis, Ellas Spyratou, Maria Karnachoriti, Maria Anthi Kouri, Ioannis Stathopoulos, Nikolaos Danias, Nikolaos Arkadopoulos, Spyros Orfanoudakis, Ioannis Seimenis, Athanassios G. Kontos, Efstathios P. Efstathopoulos

**Affiliations:** 1 2nd Department of Radiology, Medical School, National and Kapodistrian University of Athens, 11527 Athens, Greece; dimitriskalatzis@gmail.com (D.K.); spyratouellas@gmail.com (E.S.); mariakouri90@gmail.com (M.A.K.); ianstath@hotmail.com (I.S.); 2 Department of Medicine, Democritus University of Thrace, 68100 Alexandroupolis, Greece; mariakarnach@gmail.com; 3 School of Applied Mathematical and Physical Sciences, National Technical University Athens, 15780 Athens, Greece; akontos@mail.ntua.gr; 4 Medical Physics Program, University of Massachusetts Lowell, Lowell, MA 01854, USA; 5 4th Department of Surgery, School of Medicine, Attikon University Hospital, University of Athens, 12462 Athens, Greece; ndanias@med.uoa.gr (N.D.); narkadopoulos@med.uoa.gr (N.A.); 6 Alpha Information Technology S.A., Software & System Development, 68131 Alexandroupolis, Greece; spyros-orf@hotmail.com; 7 Medical School, National and Kapodistrian University of Athens, 11527 Athens, Greece; iseimen@med.uoa.gr

**Keywords:** Raman spectroscopy, colorectal cancer, tissue discrimination, machine learning, deep learning

## Abstract

Raman spectroscopy (RS) techniques are attracting attention in the medical field as a promising tool for real-time biochemical analyses. The integration of artificial intelligence (AI) algorithms with RS has greatly enhanced its ability to accurately classify spectral data in vivo. This combination has opened up new possibilities for precise and efficient analysis in medical applications. In this study, healthy and cancerous specimens from 22 patients who underwent open colorectal surgery were collected. By using these spectral data, we investigate an optimal preprocessing pipeline for statistical analysis using AI techniques. This exploration entails proposing preprocessing methods and algorithms to enhance classification outcomes. The research encompasses a thorough ablation study comparing machine learning and deep learning algorithms toward the advancement of the clinical applicability of RS. The results indicate substantial accuracy improvements using techniques like baseline correction, L2 normalization, filtering, and PCA, yielding an overall accuracy enhancement of 15.8%. In comparing various algorithms, machine learning models, such as XGBoost and Random Forest, demonstrate effectiveness in classifying both normal and abnormal tissues. Similarly, deep learning models, such as 1D-Resnet and particularly the 1D-CNN model, exhibit superior performance in classifying abnormal cases. This research contributes valuable insights into the integration of AI in medical diagnostics and expands the potential of RS methods for achieving accurate malignancy classification.

## 1. Introduction

Raman spectroscopy is a technique used to study the vibrational modes of molecules by analyzing the light that is inelastically scattered when a sample is illuminated with monochromatic light, typically from a laser source [1]. Raman spectroscopy has many potential applications in medicine, due to its ability to non-invasively analyze the molecular structure of tissues and fluids [2]. In the field of cancer research, RS has been used to diagnose various types of cancer, including breast, prostate, lung, skin, and colorectal cancer [3].

Colorectal cancer, specifically, is a type of cancer that affects the colon or rectum, which are part of the digestive system, and it is one of the most common types of cancer. Early detection and treatment of colorectal cancer can improve outcomes and increase the chances of survival. RS has been investigated as a potential diagnostic tool for colorectal cancer and similar pathologies with sensitivities and specificities usually higher than 80% in ex vivo [4,5] and in vivo studies [4,6] by using multivariate statistical techniques such as principal component analysis (PCA), linear discriminant analysis (LDA), and partial least squares regression (PLSR). Cluster analysis methods, including hierarchical clustering or k-means clustering, have also been applied in Raman spectroscopy and are commonly used in Raman imaging [7].

However, the preprocessing methods play a crucial role in the Raman spectra analysis. Recently, they used Raman spectroscopy for brain tumor identification by testing six preprocessing methods on a dataset of over 900 brain tissue samples [8]. Recently, a data analysis pipeline for Raman spectra was introduced, emphasizing its adaptability to specific applications and its three primary procedural groups: data pre-treatment, pre-processing, and modeling [9]. Another approach evaluates the statistical variability in training spectra. This method aids in selecting optimal preprocessing techniques, enhancing model performance, and ultimately establishing Raman spectroscopy as a reliable tool for noninvasive health monitoring, with potential implications for fields such as diabetes diagnosis [10]. Moreover, the effectiveness of training a convolutional neural network (CNN) using synthetic data to perform all preprocessing steps for Raman spectra has been published, including cosmic ray removal, signal smoothing, and baseline subtraction [11].

However, analyzing Raman spectra with statistical models cannot be automated and is less precise [12]. Combining Raman spectroscopy with machine and deep learning algorithms can automate the analysis process and improve the accuracy and efficiency of disease diagnosis and treatment [13]. In another study [14], researchers successfully integrated near-infrared Raman spectroscopy with support vector machines to achieve outstanding results, with the approach delivering a remarkable multi-class classification accuracy for colonic tissue specimens, highlighting its significant promise for precise colon cancer diagnosis. In another relevant study [15], Raman spectroscopy was combined with a random forest classifier to identify tumor cells infiltrating normal-appearing brain tissue. The random forest classifier achieved an accuracy of 80% in discriminating cancerous tissue from non-cancerous tissue.

Moreover, in [16], Raman spectroscopy and convolutional neural networks were used to classify breast tissue samples as benign or malignant. The CNN classifier achieved an accuracy of 92% for discriminating benign tissue from malignant tissue. In our previous study [17] we analyzed the Raman biochemical markers of colorectal cancer from 10 patients, where mainly changes in the intensities of specific collagen and protein Raman bands are evidenced. Recently, human healthy and cancerous colon specimens were surgically resected and analyzed via Raman spectroscopy. A transfer learning model, based on a one-dimensional convolutional neural network (1D-CNN), was developed and evaluated using a Raman open database, consisting of spectra from pathogen bacteria, for the pre-training process. Notably, the 1D-CNN transfer learning model achieved an accuracy of 88.7%, which is 5.3% higher compared with the non-transfer learning model, in discriminating between healthy and cancerous tissue [18].

In this study, we aimed to examine the optimal pipeline to combine AI techniques with spectra data without using data augmentation. Our decision was guided by the need for realistic data in Raman spectroscopy and the sufficient quality of our original data. To achieve this, we proposed preprocessing methods and algorithms for the best classification output of different types of malignancy including adenocarcinoma and carcinoma. We followed an extensive ablation study protocol, comparing and visualizing both machine learning and deep learning algorithms to advance the clinical potential of the RS method.

## 2. Materials and Methods

### 2.1. Patient Data Collection and Raman Spectra Acquisition

A cohort of 22 patients undergoing open colorectal surgery and 442 spectra of human colorectal tissues were collected; 221 were healthy and 221 cancerous. The patients were diagnosed with malignancies of variable anatomic regions (cecum, ascending colon, transverse colon, descending colon, sigmoid colon, and rectum), as well as variable grades and stages (Table 1). All the specimens were collected after approval by the Ethics Committee of the School of Medicine of Attikon University Hospital and written informed consent was obtained from all patients. The specimens were divided into pieces. One part was cut into 5 × 5 × 0.5 mm^3^ slices with a microtome preserved in a non-toxic zinc-based fixative (Z7) [19], which demonstrates excellent protein preservation and protection against tissue autolysis. The other part was immersed in a formalin fixative solution for histopathological examination.

Raman characterization was carried out under 785 nm excitation in the 500–3200 cm^−1^ frequency range. In brief, the laser beam was focused on random tissue spots with scattering volumes of ~34 μm^3^. Details of sample preparation and Raman acquisition sequence can be found in our previous work [17].

### 2.2. Preprocessing Steps

Raman spectra are sensitive and mostly noisy biophysical data; hence, they can be cumbersome inputs to machine and deep learning algorithms. Thorough data cleaning and preprocessing are usually needed to manipulate and extract the underlying biochemical information. The sampling frequencies of the Raman shift in our dataset are uniformly distributed. Therefore, rather than simply using the raw data, we applied consecutively preprocessing steps and examined their impact on different machine learning algorithms.

The first preprocessing step applied to the data was the baseline correction. This method subtracts a baseline signal from the raw Raman spectrum, which can be caused by scattered light or other sources of signals. In our case, the background origin is autofluorescence, which interferes with the Raman signal and makes it difficult to interpret [20]. For the subtraction of autofluorescence, we used the statistics-sensitive nonlinear iterative peak-clipping (SNIP) algorithm employing 80 iterations [21].

The second preprocessing step involved intensity normalization, which is a widely used technique in Raman spectroscopy. This step aims to remove variations in signal intensity, making the signals more comparable and facilitating their analysis. We used the Euclidean norm, also known as the L2 norm [22]. Each element in the RS vector corresponds to a specific wavelength or Raman shift, and the complete vector captures the spectral information across a range of wavelengths. Once the L2 norm is obtained, each element of the Raman spectrum vector is divided by this value. The formula for the Euclidean norm of a vector x = [*x*_1_, *x*_2_, …, *x_n_*] is:

(1) $$\left\|x\right\|=\sqrt{x_{1}^{2}+x_{2}^{2}+\ldots +x_{n}^{2}}$$

Afterward, a 1D median filter with a five-size window was used over all available spectra. A 1D median filter is a signal processing technique that is used to remove noise from a one-dimensional signal, such as a Raman spectrum.

Finally, PCA is applied to reduce the dimensionality of a dataset by identifying the directions of maximum variance in the data, known as principal components. PCA can be useful in Raman spectroscopy to identify the most important variables in a dataset, which can improve the visualization and interpretation of the data [23]. It is important to notice that PCA is an unsupervised method, which means that it does not consider the sample’s labels, thus it can be combined with other machine learning-supervised methods for classification.

### 2.3. Algorithms, Training Process, and Evaluation Methods

To find the ideal pipeline, different combinations of preprocessing methods and algorithms were investigated through our study. We trained four machine learning (ML) classification models based on logistic regression, support vector machine (SVM), random forest, and XGBoost, and two deep learning (DL) models based on convolutional neural network and residual CNN.

In our study, we employed a binary classification task to differentiate between 221 healthy and 221 abnormal spectra. Three spectra region alternations were used in this study: the low region where the frequencies from 700–1800 cm^−1^ selected the whole spectral region, the high region where the frequencies from 2800–3100 cm^−1^ were selected instead, and the combination of the previous two (low + high region). Moreover, the low region, high region, and low + high regions have 1211, 477, and 1688 dimensions, respectively. Each dimension’s value represents the Raman intensity.

Before partitioning our data for further analysis for ML models, we performed all steps on the complete spectral region of 500–3200 cm^−1^ of our preprocessing pipeline. However, when it came to deep learning models, before partitioning our data, we incorporated the initial two steps of our preprocessing pipeline. More specifically, the first two steps of the pipeline were the baseline correction and the L2 normalization. The reason we chose to implement only two preprocessing steps for our deep learning methods is as follows: Median filtering can be beneficial for machine learning, especially when dealing with data containing impulse noise or outliers, as it helps mitigate their impact on model performance. However, in deep learning, where models can automatically learn complex features, the need for median filtering is typically reduced, as these models can handle noisy data more effectively.

Regarding PCA, it can be valuable for dimensionality reduction in machine learning, which aids in simplifying complex datasets. However, in deep learning, dimensionality reduction using PCA may not always be necessary. Deep neural networks are designed to capture high-dimensional relationships in the data, often rendering PCA less advantageous.

As the final preprocessing step exclusively for our ML models, we employed PCA to conduct dimensionality reduction on the Raman spectral data. Our objective was to retain the highest classification performance. Eventually, each region (low, high, and low + high) was separately transformed into 30 principal components. After careful analysis and comparisons, we selected the low + high region as the input for our deep learning and machine learning models. Therefore, to compare the ML and DL models we used the low + high region spectra for the training and validation and the respective preprocessing steps mentioned above. 

For the comparison study, the best performance models of the ML and DL methods were trained and validated with respective preprocessing steps to three spectra region alternations (low, high, and low + high).

The machine learning methods were implemented using the scikit-learn package [24]. The training and validation were conducted using a leave-one-patient-out cross-validation approach. In this method, each patient in the dataset was held out as the validation data while the remaining patients were used for training the model. This process was repeated for each patient in the dataset, and the results were averaged to produce a single estimation. For our study, we employed a leave-one-patient-out cross-validation scheme.

Deep learning methods were developed using the Keras Application Programming Interface [25]. The architecture of the 1D-CNN consists of one input layer that specifies the shape of the input tensor and three hidden layers. The hidden layers include a 1D convolutional layer with 10 filters of size 3. An activation layer follows the convolutional layer which applies the ReLU (Rectified Linear Unit) activation function element-wise to the output of the previous layer, resulting in a tensor of the same shape. After the activation layer, a batch normalization layer normalizes the activations of the previous layer, aiding in the stabilization and acceleration of the training process. A dropout layer is applied after batch normalization, randomly deactivating a fraction of the units to prevent overfitting. The same pattern is repeated with another 1D convolutional layer, this time with 25 filters of size 3. Following the second activation layer and batch normalization layer, an average pooling layer with a pool size of 8 is used. The subsequent flattened layer reshapes the tensor into a one-dimensional vector. Finally, a dense layer with a single unit is added. This layer performs a linear transformation on the input, followed by a sigmoid activation function.

Finally, in the 1D-ResNet [26], the input sequence is passed through a series of residual blocks, each of which contains multiple convolutional layers and batch normalization layers, as well as a shortcut connection that allows the output of the block to bypass the convolutional layers. The residual connection is added to the output of the convolutional layers, which helps to preserve the original signal and reduce the loss of information during training. The deep learning model was trained for 40 epochs with a learning rate of 0.001, using the Adam optimizer algorithm and applying the binary cross entropy loss function. Similarly, in the DL models, the training and validation were performed under 10-fold cross-validation. Moreover, we employed L2 regularization with a strength of 0.0001 in our modeling approach, incorporating it into both the XGBoost model and deep learning architectures. This regularization technique was applied to mitigate overfitting and promote the generalization ability of the models.

## 3. Results and Discussion

To determine the optimal performance of the ML models, PCA was performed to low + high spectral region, resulting in 10, 20, 30, and 40 principal components (PCs) for each region. Table 2 shows the cumulative explained variances for each number of principal components.

**Figure 1:**
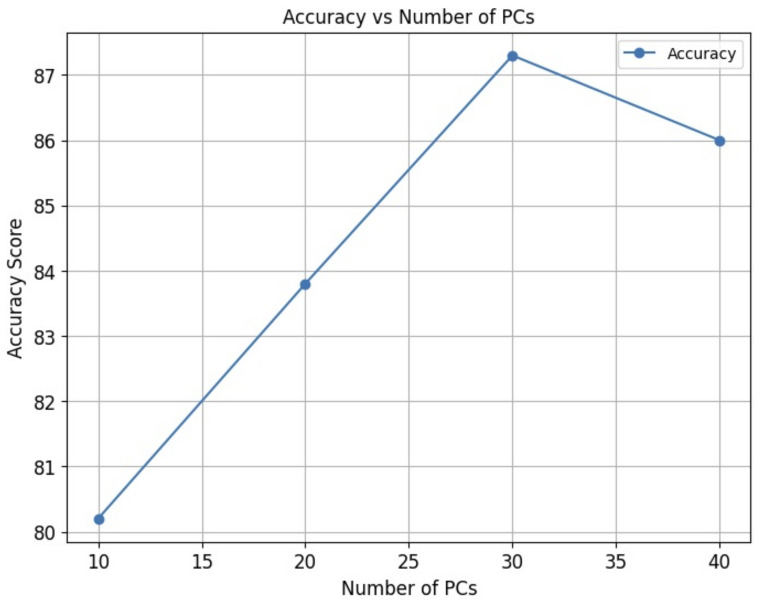
Classification results (accuracy %) of the XGBoost model applied to the low + high region with varying numbers of principal components.

By evaluating the performance of the XGBoost model with varying numbers of principal components (Figure 1), we aimed to identify the optimal number of principal components for accurate classification and discrimination of healthy and cancerous tissue samples.

Figure 1 presents the performance results of the XGBoost machine learning models using different numbers of principal components for the low + high spectral region. Specifically, using 10 PCs and 20 PCs resulted in an accuracy of 80.2% and 83.8%, respectively. Notably, the higher accuracy of 87.3% was reached by using 30 PCs while with 40 PCs the accuracy decreased to 86%.

These results suggest that the choice of the number of principal components can significantly impact the performance of the XGBoost models. Based on these findings, the low + high region with 30 principal components demonstrated the highest accuracy for classifying healthy and cancerous tissue samples. Therefore, to comprehensively compare the performance of different ML models, we utilized the low + high region with 30 PCs as the input for our analysis. By applying this consistent input across all ML models, we aimed to establish a fair and objective comparison of their classification abilities.

**Figure 2:**
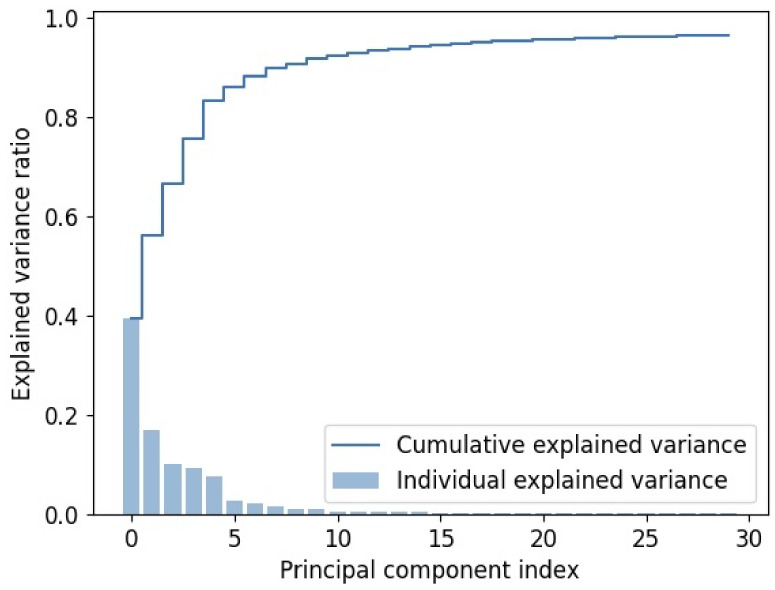
Explained variance plot of low + high region with 30 principal components.

The variance plot (Figure 2) presents the cumulative explained variance which reached 96.4% and the individual explained variance of the low + high region with 30 PCs. The plot displays the principal component index on the x-axis and the explained variance ratio on the y-axis. It reveals that the first few PCs capture a significant portion of the variance, with diminishing returns observed as more PCs are included. This indicates that a low portion of variance PCs can effectively capture the essential information present in the spectral data. Thus, the selection of 30 PCs is the appropriate choice for achieving a balance between dimensionality reduction and maintaining sufficient explained variance for accurate classification of healthy and cancerous tissue samples.

**Figure 3:**
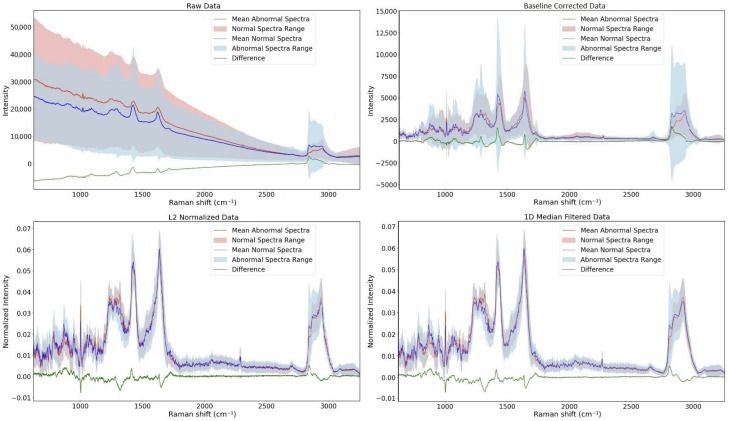
Mean spectra of both normal and abnormal classes and their difference. (**top left**): Raw spectra, (**top right**): spectra after baseline correction, (**bottom left**): spectra after applying L2 normalization, and (**bottom right**): spectra after applying 1D Median Filter.

The preprocessing steps produce different spectra with various characteristics. In all four images, the average spectra of normal and abnormal specimens and their subtraction are depicted. The main variations are shown in Figure 3, bottom right. The highest differences were observed at 866, 1005, 1342, 1437, 1671, 1748, 2852, 2890, 2935, and 2974 cm^−1^. These results strongly align with the outcomes of our previous work, which involved a biochemical analysis of these differences [14]. The current results deriving from double the amount of data confirm our previous differences in intensity.

To further highlight the impact of preprocessing steps on models’ performance, we picked as a reference the XGBoost algorithm, and its training evaluation was performed in the low + high region. Subsequently, we tracked the accuracy and recall of each sequential preprocessing step to determine their effects.

**Figure 4:**
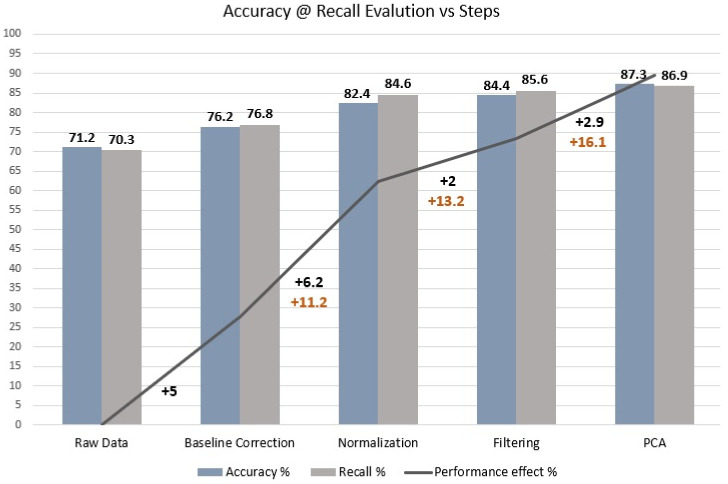
Plot of the percentage improvement in the accuracy and recall metrics of the XGBoost classifier after each preprocessing step.

The results are presented in Figure 4. The baseline correction, the L2 normalization, the filtering, and the PCA offer a boost in accuracy of 5%, 6.2%, 2%, and 2.9%, respectively. Overall, the improvement in accuracy and recall reached a total of 16.1% and 16.6%, respectively.

In Table 3, we present a side-by-side comparison of our proposed preprocessing pipeline with three reference pipelines [9,10]. The assessment is grounded in the measured enhancements in accuracy and recall achieved by each pipeline.

The best performance combinations of the reference pipeline [9], which incorporates (a) Savitzky–Golay smoothing, SNIP baseline correction, standard normal variate (SNV), and PCA, achieved an accuracy improvement of 15.1% and a recall improvement of 19.7%. Additionally, the configuration of (b) Gaussian smoothing, SNIP baseline correction, standard normal variate (SNV), and PCA achieved an accuracy improvement of 14.8% and a recall improvement of 17.3%. 

Another reference [10], which employed (c) SNIP baseline correction, SNV, and Savitzky–Golay smoothing, achieved an accuracy improvement of 13.6% and a recall improvement of 13.5%.

The comparison results of the four different machine learning algorithms and two deep learning models are shown in Table 4. Among the ML models, the XGBoost algorithm presents the best performance across all metrics, achieving an accuracy of 87.3%. Specifically, both the XGBoost and random forest ML models exhibit excellent performance when classifying normal tissues, while the SVM and logistic regression models show greater effectiveness in identifying abnormal tissues.

**Figure 5:**
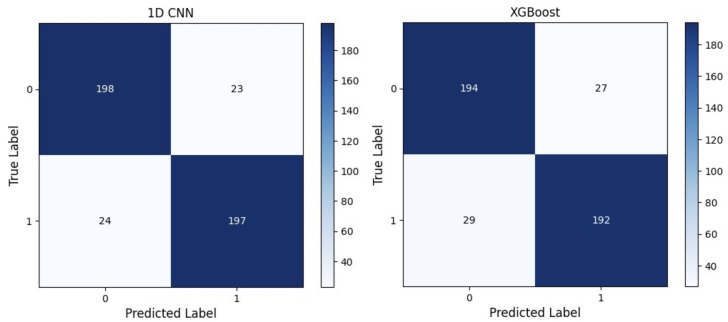
Confusion matrix: 1D-CNN and XGBoost. The labels 0 and 1 correspond to healthy and cancerous tissues, respectively.

In contrast, the DL models excel primarily over ML models in classifying abnormal cases, with the 1D-CNN model showcasing the best results in both classes, achieving an accuracy level of 91.4% and an essential recall rate of 87.8% and 95% for normal and abnormal cases, correspondingly (Figure 5). The recall rate holds immense importance because a false-negative assessment generated by the deep learning model in clinical applications can provoke fatal consequences for the patient.

Then, a more detailed analysis was carried out on the best-performed DL and ML models by examining their performance not only in the low + high region as mentioned above but also in the rest two regions (low, high). For the DL model, we performed the first two preprocessing steps as mentioned above and for the ML model, all preprocessing steps were used. In the last step, where the PCA took place, 30 PCs were used.

As Table 5 shows, both models produce low performance when the high region of the spectra is used, with 78.7% and 83.7% accuracy for the XGBoost and 1D-CNN models, respectively. On the other hand, the low region produces comparable results with the full spectra experiments, with XGBoost reaching an 84.8% accuracy and the 1D-CNN a 90.2% accuracy level. The combination of the two subregions boosts the accuracy of the XGBoost model to 87.3% and the 1D-CNN’s accuracy to 91.4%.

**Figure 6:**
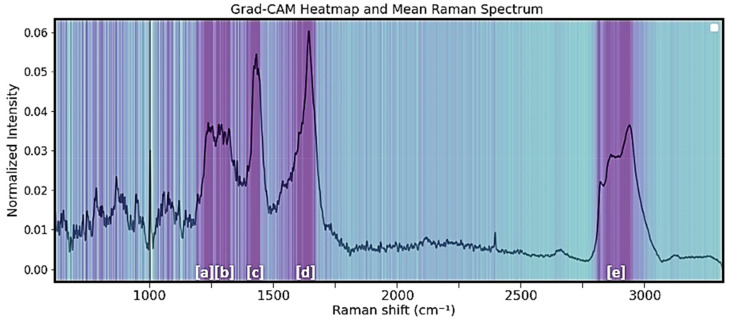
Importance bands over the whole spectra region of the 1D CNN using Grand-CAM (dark purple represents areas of higher importance and [a]–[e] the spectral regions).

Figure 6 provides a visual representation via gradient-weighted class activation mapping (Grand-CAM) [27] of the varying levels of importance or activation of features within a 1D CNN model when applied to Raman spectra data. This heat map helps in interpreting the predictive capabilities of the model by highlighting the regions of the input spectra that contribute the most to the final prediction. Spectral regions that mostly affect the decision making of the 1D-CNN model are the regions of 1210–1280 cm^−1^, 1290–1350 cm^−1^, 1410–1472 cm^−1^, 1617–1687 cm^−1^, and 2840–2945 cm^−1^ (regions [a]–[e] in Figure 6).

## 4. Discussion

In this current study, we embarked on a comprehensive examination of the optimal pipeline for integrating AI techniques with spectral data, all without resorting to data augmentation methods. Our primary objective was to develop and refine preprocessing methods and algorithms that would yield the most accurate classification outcomes for different types of malignancies, specifically adenocarcinoma and carcinoma. To achieve this, we diligently followed an extensive ablation study protocol, meticulously comparing and visually representing the performance of both machine learning and deep learning algorithms. This systematic investigation was undertaken with the overarching aim of advancing the clinical potential of the Raman spectroscopy method in the field of oncology. By optimizing the fusion of AI and spectral data and rigorously evaluating the performance of various algorithms, we sought to contribute valuable insights and methodologies that could enhance the accuracy and reliability of malignancy classification, ultimately benefiting clinical practice and patient care. Our findings underscore the importance of preprocessing steps in improving classification outcomes. Through preprocessing spectral analysis techniques like baseline correction, L2 normalization, filtering, and PCA, we achieved a remarkable 16.1% and 16.6% enhancement in accuracy and recall, respectively. These enhancements not only contribute to the overall accuracy but also hold significant clinical implications, reducing the risk of misclassification. The comparison between machine learning and deep learning algorithms revealed the strengths of each approach. Machine learning models, specifically XGBoost and random forest, demonstrated their effectiveness in classifying both normal and abnormal tissues. Deep learning models, notably the 1D-CNN model, excelled in identifying abnormal cases, with an accuracy rate of 91.4% and an essential recall rate of 95%. The emphasis on recall rate in deep learning is particularly crucial in clinical applications, where false negatives can have severe consequences. Furthermore, a detailed analysis of the model’s performance in different spectral regions revealed interesting insights. While the high spectra region (2800–3100 cm^−1^) yielded lower accuracy, the combination of low (700–1800 cm^−1^) and high (2800–3100 cm^−1^) regions significantly boosted the accuracy of both XGBoost and 1D-CNN models, highlighting the importance of considering multiple spectral regions in RS-based malignancy classification. These findings open doors for further research and development in the field, with the ultimate goal of improving patient outcomes and advancing the clinical application of RS-based diagnostics. Our attention to preprocessing techniques to enhance classification outcomes aligns with previous research highlighting the critical role of data preprocessing in spectroscopy-based studies. Various studies have underscored the significance of techniques such as baseline correction, normalization, and feature extraction in improving the quality of spectral data and subsequently enhancing classification accuracy [8,9,10]. However, our study stands out by demonstrating substantial enhancements of 16.1% in accuracy and 16.6% in recall in colorectal abnormality classification, underscoring the practical importance of these techniques in clinical applications. These results complement the existing literature by providing empirical evidence of the effectiveness of specific preprocessing steps in the context of malignancy classification. The comparison between machine learning and deep learning models mirrors ongoing discussions in the field of artificial intelligence and medical diagnostics. Prior research has explored the advantages and limitations of both paradigms in various healthcare applications [10,11,12,13,14]. Our findings align with the consensus that machine learning models, such as XGBoost and random forest, are well suited for tasks requiring interpretability and robust performance on spectral datasets, as demonstrated in their effectiveness in classifying both normal and abnormal tissues. Deep learning models, particularly the 1D-CNN model, reaffirm their strength in handling complex data patterns, as evident in their remarkable accuracy and recall rates for identifying abnormal cases. The consideration of multiple spectral regions in RS-based malignancy classification echoes our previous research [15] advocating for the importance of spectral region selection. The majority of these regions correspond to Raman bands with different normalized intensities between cancerous and normal tissues. In particular, the protein Raman bands located at 1330 cm^−1^ and 1658 cm^−1^ show differences between normal and cancer spectra that have been attributed to protein overexpression in cancer tissues. Furthermore, the differences in the Raman modes at 1250 cm^−1^ and 1450 cm^−1^ have been attributed to variations in collagen and lipid content, respectively. In addition, the importance of the high-wave region in the classification task was stated. This spectral region is characterized by sharp differences that are mainly due to the higher lipid-to-protein ratio in normal tissues based on the operating intensities at 2852 cm^−1^ and 2935 cm^−1^. This alignment greatly enhances the reliability of the DL algorithm results. In summary, our research not only advances the clinical potential of RS-based diagnostics but also enriches the existing body of knowledge in the domains of spectroscopy, medical diagnostics, and machine learning. The synergy between our findings and the established literature forms a foundation for continued exploration and innovation in the pursuit of improved patient outcomes and the broader adoption of RS-based diagnostic methodologies.

## 5. Conclusions

In conclusion, our study represents an advancement in the field of medical diagnostics, particularly in the context of colorectal cancer classification using Raman spectroscopy (RS) data and AI techniques. Through rigorous preprocessing methods, a comparative analysis of machine learning and deep learning models, and insightful exploration of spectral regions, we have contributed valuable insights and methodologies. These findings not only enhance the accuracy of malignancy classification but also hold the potential to positively impact clinical practice. As we conclude this research, we look forward to further developments that will continue to advance the clinical application of RS-based diagnostics and, ultimately, improve patient outcomes.

## Figures and Tables

**Table 1:** The clinical data of the patients regarding gender, age, anatomic region, stage, and grade of cancer.

Samples	Gender	Age	Anatomic Region	Type of Malignancy	Stage	Grade
1.	Male	69	Rectum	Adenocarcinoma	pT3N1	G2
2.	Male	56	Sigmoid colon	Carcinoma	pT3N2b	G3
3.	Female	77	Hepatic flexure	Adenocarcinoma	pT3N0	G3
4.	Male	74	Rectosigmoid	Adenocarcinoma	pT3pN0	G1
5.	Male	66	Orthosigmoid	Adenocarcinoma	ypT3N2b	G2
6.	Male	61	Hepatic flexure	Adenocarcinoma	pT4bN0	G2
7.	Male	76	Caecum	Adenocarcinoma	pT2N0	G2
8.	Female	56	Rectum	Adenocarcinoma	ypT3N2a	G2
9.	Male	69	Rectum	Adenocarcinoma	pT4bN1	G2
10.	Male	77	Hepatic flexure	Adenocarcinoma	pT2N0	G2
11.	Female	49	Transverse colon	Adenocarcinoma	pT3N1M1	G2
12.	Male	56	Transverse/descending colon	Adenocarcinoma	pT3N0	G1
13.	Male	75	Sigmoid colon	Adenocarcinoma	pT3N1c	G2
14.	Male	66	Ascending/transverse coon	Adenocarcinoma	pT2N0	G2
15.	Female	50	Caecum	Adenocarcinoma	pT1N0	G1
16.	Male	74	Sigmoid colon	Adenocarcinoma	pT3N0	G2
17.	Male	87	Sigmoid	Adenocarcinoma	pT3N0	G2
18.	Male	85	Sigmoid	Adenocarcinoma	pT2N0	G2
19.	Male	87	Sigmoid	Adenocarcinoma	pT3N0Mx	G2
20.	Male	76	Transverse colon	Adenocarcinoma	pT4bN0	G2
21.	Male	63	Transverse colon	Adenocarcinoma	pT3N0M1	G2
22.	Male	77	Sigmoid	Adenocarcinoma	pT3N2a	G2

**Table 2:** Cumulative explained variances (%) for the low + high region, with different numbers of principal components used in the analysis.

Principal Components	Cumulative Explained Variances (%)
10	91.6
20	95.3
30	96.4
40	96.9

**Table 3:** Detailed classification results using the XGBoost model for four preprocessing pipelines.

	Precision	Recall	F1-Score	Accuracy
A pipeline	89.2	82.5	85.7	86.3
	83.7	90	86.8	
B pipeline	87.3	84.4	85.8	86
	84.9	87.7	86.2	
C pipeline	84.2	85.8	85	84.8
	85.5	83.9	84.7	
Suggested pipeline	87	87.8	87.4	87.3
	87.7	86.9	87.3	

**Table 4:** Detailed classification results of four ML and two DL models for both normal and abnormal classes.

	Class	Precision	Recall	F1-Score	Accuracy
**Machine Learning Models**					
Logistic regression	Normal	77.4	62	68.8	71.9
	Abnormal	68.3	81.9	74.5	
SVM	Normal	85.6	83.7	84.7	84.8
	Abnormal	84.1	86	85	
Random forest	Normal	84.8	88.2	86.5	86.2
	Abnormal	87.7	84.2	85.9	
XGBoost	Normal	87	87.8	87.4	87.3
	Abnormal	87.7	86.9	87.3	
**Deep Learning Models**					
1D-CNN	Normal	94.6	87.8	91.1	91.4
	Abnormal	88.6	95	91.7	
1D-Resnet	Normal	94.2	87.8	90.9	91.2
	Abnormal	88.6	94.6	91.5	

**Table 5:** Detailed classification results (weighted average metrics) for the best ML and DL model at the three alternations of wavenumber regions.

	Model	Precision	Recall	F1-Score	Accuracy
Low + high region	XGBoost	87.6	86.8	87.2	87.3
	1D CNN	88.6	95	91.7	91.4
Low region	XGBoost	85.6	83.7	84.6	84.8
	1D CNN	88.5	92.3	90.4	90.2
High region	XGBoost	78.9	78.2	78.6	78.7
	1D CNN	83.1	84.6	83.8	83.7

## Data Availability

Data underlying the results presented in this paper are not publicly available at this time but may be obtained from the corresponding author upon reasonable request.
